# Creating a digital segmentation tool to tailor weight management advice: the Diet Types Survey

**DOI:** 10.3389/fpsyg.2025.1611946

**Published:** 2026-01-09

**Authors:** E. Brindal, G. A. Hendrie

**Affiliations:** Commonwealth Scientific and Industrial Research Organisation (CSIRO), Health & Biosecurity, Adelaide, SA, Australia

**Keywords:** dieting, eating, implementation, obesity, segmentation

## Abstract

**Background:**

Tailoring of weight management interventions has tended to focus on demographic characteristics, anthropometric data, health behavior, but seldom on psychological variations or personality traits. The objectives of this study were to (1) shorten and validate an existing dieting style tool; and (2) launch an online tool to assess the uptake and acceptability of segmented feedback.

**Method:**

This study used an existing dieting styles tool consisting of over 100 items about personality traits, self-efficacy, restraint, impulsivity, perfectionism, food involvement, motivation, mindfulness, habits, and habits related to eating and dieting behavior. The number of items was reduced, and a shorter survey was tested in a sample of 1,551 Australians (83.2% female). The revised version with a blurb based on different styles was beta tested (*n* = 74) and launched as the Diet Types tool on the homepage of an online weight management program.

**Results:**

The structure of the short form survey replicated the original with both Eigenvalue criteria and Monte Carlo analysis, indicating the presence of 5 factors that would become the final types of Thinker, Craver, Socializer, Foodie, and Freewheeler. The correlation between factors ranged from 0.265 to 0.506. Internal consistency was acceptable to excellent. The launch of the final Diet Types tool resulted in 29,394 participants completing the survey in 5 days. The sample was mostly women (81.3%), with 42.9% aged 30–49 years and 36.0% aged 50–69 years. Three-quarters of the sample was categorized as overweight or obese (41.9% obese). One-third of the sample (33.8%, *n* = 9,935) was classified as having a single dominant type, with an additional 38.5% (*n* = 11,310) showing two equally dominant options. Thinker was the most common (29.7%), followed by Craver (18.1%). Those classified as Cravers were more highly represented in the obese weight status category and reported the highest number of dieting attempts.

**Conclusion:**

The development of a short segmentation tool with feedback about dieting personality proved to be popular, with high uptake particularly among older females living with obesity within the Australian population. In future, the information collected may help to create and deliver messages that are more relevant and persuasive than generic messages for weight management.

## 1 Introduction

Obesity in adults has more than doubled since 1990, with 43% of adults worldwide classified as overweight or obese (in 2022). The adverse health risks associated with obesity have also increased, along with the social and economic impacts, which are expected to cost USD$3 trillion by 2030 if nothing can be done to address this global public health challenge (World Health Organization (WHO), 2023). The World Health Organization has released an accelerated plan to stop obesity, and consistent with this, the goal of Australia’s National Obesity Strategy is “to halt the rise and reverse the trend in the prevalence of obesity in adults by 2030” (Commonwealth of Australia, 2022). To achieve such an ambitious target, people will require better access to tailored support to change their behavior and reduce their risk of weight gain and obesity (Commonwealth of Australia, 2022). Tailored interventions involve a level of segmentation to deliver interventions to one specific individual or group of individuals with similar characteristics. These segments can be based on individuals’ characteristics, needs, preferences, and/or situation. Tailored interventions can be more effective in changing behavior and achieving weight loss (Lustria et al., 2013; Napolitano et al., 2021; Noar et al., 2007).

Tailoring of weight management interventions has tended to be based on demographic characteristics such as age or gender; anthropometric data such as baseline weight; health goals or health behavior such as levels of physical activity or eating habits (Neville et al., 2009; Ryan et al., 2019); but seldom on psychological variations or personality traits. Yet, evidence suggests that different personality characteristics may be associated with preferences for different dieting approaches and success (Munro et al., 2011; Sitton and Weber, 1987). A deeper understanding of psychological traits that relate to dieting behavior and eating habits is needed. Such knowledge may help to create and deliver messages targeted to specific segments that are more relevant, informative, and persuasive than generic messages, and could be useful in providing more effective advice within weight management programs.

In previous work, an assessment tool was developed to describe different types of dieters, with the intent of using it to provide tailored advice within a digital weight loss program. The tool included questions on personality traits, self-efficacy, restraint, impulsivity, perfectionism, food involvement, motivation, mindfulness, habits, and habits related to eating and dieting behavior. The initial measure was refined through factor analysis into five core diet styles: Foodie, Temptation, Overthinking, Social, and Impulsive. Construct validity appeared promising, with each factor correlated with weight-related behavior such as diet quality, self-reported health, and Body Mass Index in the expected direction.

The initial objective of this work was to identify different psychological profiles that could be used to tailor program content to suit different dispositions with the longer-term aim of changing program engagement and improving retention. People who stay engaged longer with weight loss programs tend to have more favorable longer-term outcomes (Hendrie et al., 2025). Tailoring in this sense could be described as creating segmentation to enhance messaging acceptance or the level of processing (Hawkins et al., 2008). Feeling more connected to intervention materials has been shown to promote improved behavioral intention for weight loss (Kreuter et al., 1999). To achieve these longer-term ambitions, the current focus was to capture these diet styles in an engaging tool. The aims of this tool were to be able to segment different people into relevant groups, to be integrated into an existing program, and to provide information to people to assist them in understanding their own diet styles and how they may relate to weight management and eating. After factor analysis, a tool to assess the original styles would have included over 100 items (Brindal and Golley, 2021), making it potentially less engaging and less suitable for application in a consumer-facing program. Therefore, the objectives of this study were to (1) shorten and validate the dieting style tool; and (2) launch the online tool to assess the uptake and acceptability of tailoring dieting style advice to users within the context of a real-world weight loss program.

## 2 Development of short form, validation, and beta testing (Study 1)

Research has shown that longer surveys receive lower participation and completion rates. For example, Galesic and Bosnjak (2009) reported that 75% of people commenced a 10-min survey compared to 63% of people for a 20-min survey. Furthermore, 68% completed all items in the 10-min survey compared to 57% for the 20-min survey. The ability to combine existing scales into a single questionnaire is also likely to be beneficial in terms of minimizing measurement error. Alwin and Beattie (2016) report that batteries of questionnaires are less reliable than a series of questions. Therefore, streamlining and shortening existing scales could benefit participant engagement and measurement accuracy. Maloney et al. (2011) also explored whether strategic survey reduction could be done without compromising on validity. They reduced items through assessing items with the highest loading on factors for scales in existing published literature, with the aim of retaining at least 4 items for each construct. The resulting shortened scales showed acceptable validity.

The previously developed long-form Diet Styles Survey included items from different measures of a range of constructs around dieting and personality traits. The survey included more than 100 items presented across varying response formats. As one of the aims of the tool was to integrate it into an existing program, it was decided that a short tool would be more appropriate with less likelihood to impede engagement. Therefore, the aim of the first study was to reduce the number of items in the Diet Styles Survey and retain the essence of the survey and original constructs.

### 2.1 Method

Developing a short-form Diet Styles Survey involved several phases: re-analyzing the initial data collected, refining the delivery of the tool, and collecting new data from a validation and beta sample. Each is described further below.

#### 2.1.1 Secondary data modeling (Phase 1)

Items from each of the strongest loading subscales of the initial diet styles were factor analyzed to assess those items contributing most strongly to each construct. This included scale items from the original Cravings Scale, Varied Eating Scale, Behavioral Inhibition Scale, Social Closeness Subscale, and impulsivity (Brindal and Golley, 2021). The aim was to identify the 5 strongest items representing each construct. Where the strongest loading subscale included five or fewer items, factor analysis was performed to confirm that the items adequately represented the constructs. A forced 1-factor solution was used in all instances for each of the identified diet styles.

#### 2.1.2 Validation in novel sample (Phase 2)

To assess the validity of the short form survey to identify diet styles, the survey was revised following the secondary data modelling described above, and for the ease of responding, all items were presented with a standard 5-point agreement rating.

The short form survey (Table 1) was administered to a convenience sample recruited via email to a mailing list associated with a weight management website owned by Digital Wellness (formerly known as SP Health). Only those who had consented to being contacted for future research were emailed. Prior to starting the survey, participants were presented with information on the study and given the chance to consent. No incentive was offered for participation. This aspect of the study was approved under the same ethical application as the original study (CSIRO LR23/2016) (Brindal and Golley, 2021).

**Table 1:** Rotated maximum likelihood models for Diet Styles reduced items across 3 distinct samples.

Original items		Amended wording	Original data	Second sample	Launch
Varied eating patterns [foodie]		Foodie	*n* = 1,056, *α* = 0.823	*n* = 1,551, *α* = 0.760	*n* = 29,394, *α* = 0.777
1	New meal ideas are important to me	New meal ideas excite me	0.805	0.665	0.678
2	I like to try new meals	I like to try meals I have never eaten before	0.798	0.727	0.703
3	It is important for me that I have a variety of meals each month	I love having a variety of different meals each month	0.756	0.668	0.747
4	I like to eat pretty much the same foods every week^b^		−0.655	−0.613	−0.628
5	I would have the same meal every Sunday night if I could^b^	I would happily have the same meal every Sunday night	−0.511	−0.451	−0.495
Cravings [temptation]		Craver	*n* = 1,066, *α* = 0.914	*n* = 1,551, *α* = 0.908	*n* = 29,394, *α* = 0.848
1	Once I start eating, I have trouble stopping		0.832	0.654	0.556
2	If I give in to a food craving, all control is lost		0.828	0.778	0.677
3	If I am craving something, thoughts of eating it consume me		0.820	0.934	0.915
4	Whenever I have a food craving, I keep on thinking about eating until I actually eat the food		0.797	0.922	0.856
5	I have no willpower to resist my food craving	Item removed	0.792	0.831	–
Impulsivity [impulsive]		Freewheeler	*n* = 1,139, *α* = 0.691	*n* = 1,551, *α* = 0.797	*n* = 29,394, *α* = 0.771
1	I do things without thinking^a^		0.564	0.422^a^	0.517
2	I concentrate easily^b^		−0.556	−0.285	−0.308
3	I am a careful thinker^b^		−0.548	−0.434	−0.476
4	I act “on impulse”		0.540	0.955	0.888
5	I act on the spur of the moment		0.535	0.923	0.863
Social closeness [social]		Socializer	*n* = 1,138, *α* = 0.602	*n* = 1,551, *α* = 0.622	*n* = 29,394, *α* = 0.649
1	I am a warm person	Compared to others, I a warm person	0.674	0.680	0.582
2	I am sociable	I am a highly sociable person	0.610	0.690	0.708
3	I value the relationships I have with others	The relationships I have with others are a priority for me	0.478	0.491	0.601
BIS [overthinking]		Thinker	*n* = 1,233, *α* = 0.793	*n* = 1,551, *α* = 0.818	*n* = 29,394, *α* = 0.803
1	I feel pretty worried or upset when I think or know somebody is angry at me		0.721	0.738	0.673
2	If I think something unpleasant is going to happen I usually get pretty worked up		0.704	0.762	0.765
3	I worry about making mistakes		0.685	0.745	0.717
4	Even if something bad is about to happen to me, I rarely experience fear or nervousness^b^		−0.623	−0.539	−0.562
5	Criticism or scolding hurts me quite a bit		0.603	0.679	0.644

^a^Cross loading 0.264 with temptation.

Changes to the instrument are noted in the table.

b Reverse coded item.

In addition to completing the short form survey, the participants completed questions on their sociodemographic characteristics (sex, year of birth, state/territory of residence, postcode, ethnicity, occupation, height, and weight), concern and success over managing weight (7-point scale), frequency of fast food/takeaway consumption, and lifetime weight management attempts. The survey link was made available for 5 days and took 5–10 min to complete.

Factor analysis using the Maximum Likelihood Extraction method with Promax rotation was used to assess the structure of the short survey. As well as using factor analysis, histograms were inspected to assess item performance. Participants were classified into one of five dominant styles based on the rules established in the original study (Brindal and Golley, 2021). Comparisons between those with a single dominant diet style were made using Chi-square or ANOVA.

#### 2.1.3 Development and beta testing of new interface (Phase 3)

The survey was translated into a web page that presented participants with visual feedback about their diet style and specific dominant style. Short blurbs were written that summarized the dispositions associated with the different styles based on the observations in the pilot data. A small sample of subscribers to a weight management program (*n* = 100) was sent a live version of the fully active website. In addition to completing the short form survey, these participants were shown their dominant category and had the opportunity to agree/disagree. If they disagreed, they had the option to select the diet style they felt was more appropriate and to provide feedback about this decision.

### 2.2 Results

#### 2.2.1 Initial item selection and data modelling (Phase 1)

The strongest loading survey items are displayed in Table 1. Except for one of the items assessing social closeness, all the strongest five loadings were above 0.4, representing good association with the primary construct (Stevens, 1992). Internal consistency was good to excellent for Foodie, Cravings, and Overthinking, and acceptable for Impulsivity and Social Closeness. Scores based on the shortened survey were strongly correlated with scores from the full scales: Foodie (*r* = 0.682, *p* < 0.01); Temptation (*r* = 0.874, *p* < 0.01); Impulsivity (*r* = 0.785, *p* < 0.01); Social Closeness (*r* = 0.813, *p* < 0.01); Overthinking (*r* = 0.776, *p* < 0.01).

#### 2.2.2 Validation results (Phase 2)

A total of 1,551 people responded to the email to complete the short form Diet Styles Survey. A majority of the sample were female (83.2%), with a mean age of 51.6 years (range 18–90 years). One in four described their occupation as retired (24.4%), and three in four identified as Anglo-Saxon (73.6%). Participants resided primarily in New South Wales (26.2%), Victoria (22.1%), and Western Australia (21.4%). Of all respondents, 1,479 (95%) provided valid height and weight values. The mean Body Mass Index for this group was 27.1 (range 15–58). Approximately half the sample were in the normal weight classification (46.0%), and one-quarter in each of the overweight (27.2%) and obese categories (26.8%).

The factor structure of the short form survey was clean. Both the Eigenvalue criteria and the Monte Carlo analysis confirmed the presence of 5 factors. Factors 1 and 2 were moderately correlated (*r* = 0.381), as were Factors 1 and 5 (0.506). Factors 4 and 5 were weakly correlated (*r* = 0.265). Items loaded cleanly onto these factors, with only a single impulsive item having a weak cross-loading (Table 1). The internal consistency of the scales was also acceptable to excellent (Table 1).

Closer examination of responses found that some individual items had high agreement. To reduce the high proportion of people agreeing with the items from the scales constructed by the authors (Foodie and Social), changes were made to the wording (see Table 1). Due to their previous validation, the wording of the Craving and Behavioral Inhibition Scale items was not modified. One craving item was removed due to very low levels of agreement (63.9% disagreed), which skewed this item.

In examining diet styles, it was found that 32.3% (*n* = 501) of the sample were classified as having a single dominant diet style according to the original algorithm. In this sample, the most common dominant style was Foodie (36.7%), followed by Overthinking (30.7%) and Social (24.8%). Temptation and Impulsive were less frequent as a dominant style with 7.4% and <0.5% of the sample, respectively, being classified into these categories. The sample of people with a single dominant diet style (*n* = 501) was compared for the lifestyle behaviors and outcomes assessed (Table 2). The group with Impulsive as their dominant style was excluded from these comparisons due to the small cell sizes.

**Table 2:** Sample classified as having single dominant Diet Style (*n* = 501) compared by Style for Body Mass Index (BMI), gender, age, concern overweight, lifetime dieting history and frequency of fast food consumption.

Variable	Overthinking	Temptation	Foodie	Social	Total
BMI category *χ*^2^=18.84, *p* = 0.004					
Normal, *n* (%)	74 (31.6)	7 (<1%)	93 (39.7)	60 (25.6)	234 (49.1)
Overweight, *n* (%)	35 (26.5)	12 (9.1)	46 (34.8)	39 (29.5)	132 (27.7)
Obese, *n* (%)	35 (31.5)	16 (14.4)	37 (33.3)	21 (18.9)	111 (23.2)
Total, *n* (%)	144 (30.2)	35 (7.3)	176 (36.9)	120 (25.2)	477 (100)
What is your gender? *χ*^2^=5.96, *p* = 0.113					
Male, *n* (%)	19 (22.9)	3 (<1)	37 (44.6)	23 (27.7)	83 (16.6)
Female, *n* (%)	135 (32.3)	34 (8.1)	147 (35.2)	101 (24.2)	418 (83.4)
Total, *n* (%)	154 (30.7)	37 (7.4)	184 (36.7)	124 (24.8)	501 (100)
Participant age (years), *F* (3,495) = 5.56, *p* = 0.001					
Mean (SD)	48.51 (15.55)	47.84 (13.58)	54.14 (13.61)	53.64 (16.03)	51.81 (15.05)
Concern with watching weight (/7), *F* (3,495) = 11.75, *p* < 0.001					
Mean (SD)	5.30 (1.58)	6.32 (0.91)	4.80 (1.41)	4.94 (1.71)	5.10 (1.56)
Perceived success at manage weight (/7), *F* (3,495) = 15.83, *p* < 0.001					
Mean (SD)	4.29 (1.76)	2.97 (1.59)	4.62 (1.63)	5.06 (1.70)	4.51 (1.76)
Lifetime weight management history, *F* (3,495) = 17.10, *p <* 0.001					
Mean (SD)	2.03 (1.68)	3.16 (1.57)	1.43 (1.30)	1.54 (1.39)	1.77 (1.54)
Frequency of eating fast food and/or takeaways, *F* (3,495) = 10.94, *p* < 0.001					
Mean (SD)	3.77 (2.41)	4.16 (2.51)	2.72 (2.05)	2.69 (2.05)	3.14 (2.26)

Note underweight category not displayed due to low cell sizes.

Dominant styles differed by age, weight status, and lifestyle behaviors but not sex. According to post-hoc comparisons, those people with Foodie or Social dominant styles were older than those with an Overthinking dominant style. Those with Temptation as a dominant had a higher frequency of obesity, higher levels of concern for weight, and lower feelings of success related to their weight management compared to people with the other dominant styles. Those dominant in Overthinking had higher concern for weight than those with Foodie dominant traits, but lower feelings of success than with a Social dominant style. In line with these observations, Temptation-dominant people also had a higher frequency of weight management attempts than all other styles. Overthinking-dominant people had more attempts at weight management than Foodie-dominant and Social-dominant. Finally, people with Foodie-dominant and Social-dominant styles had significantly lower frequency of fast-food consumption than all other groups.

#### 2.2.3 Beta testing feedback (Phase 3)

A sample of 74 (84% women) of 100 invited people accessed the live website and provided feedback on the short form Diet Styles Survey and dominant style assignment. Most participants (81.1%) were happy with the dominant style they were presented with (selected “Yes” to “Does this sound like you?”). An additional 6.8% were explicitly not happy, and 12.2% did not respond to this question. After excluding non-responses, there was 92.3% agreement with the dominant diet style assignment.

In this sample of beta testers, 21.6% of respondents had one dominant style. Social, Foodie, and Overthinking styles were again the most dominant styles. Few people were classified as Impulsive-dominant, with average agreement scores also low for these items (Table 3). The Temptation style had the greatest variation in scores.

**Table 3:** Scores and frequency for the short form Diet Styles Survey based on beta testing with 74 subscribers to a weight management website.

Style	Frequency		Scores			
	*n*	%	Min	Max	Mean score	SD
Temptation	9	12.2	24	100	62.43	19.76
Foodie	16	21.6	36	100	75.62	12.74
Impulsive	3	4.1	24	84	53.03	12.77
Social	22	29.7	33	100	75.24	15.05
Overthinking	20	27.0	36	100	75.41	15.24
Unknown	4	5.4	nil	nil	nil	nil

Of the 5 people who disagreed with their dominant style, 4 selected Overthinking and 1 Social. Two of those who disagreed selected the same dominant category they were presented with (Overthinking). As they could not see all the descriptions prior to disagreeing, they may have felt that there were better options, but then discovered that this style was the most suitable.

### 2.3 Conclusions from the validation and beta testing

The structure of the Diet Styles Survey was replicated using the short form tool, and the internal consistency of the shortened items appeared good in the validation sample (Phase 2).

The decision to reduce items was based on the desire to improve engagement, but this choice needed to be balanced with the potential loss of complexity within the survey. The shortened items were strongly associated with the full constructs in Phase 1, but these correlations suggest some loss of depth in terms of capturing the full construct. The field of Human–Computer Interaction has explored many ways to improve engagement beyond length reduction (Cruz-Benito et al., 2017), and there may have been alternative ways to improve engagement while retaining greater depth within the survey. Associations between different styles and a variety of individual characteristics and lifestyle behaviors were consistent with those observed in the original study, which provides some reassurance that any loss of depth in measuring the constructs did not change the ability of the tool to reflect different individual traits. For example, weight and weight-related perceptions were higher for those with a dominant Temptation style compared to other groups.

One in five people was classified as having a single dominant style, which was slightly lower than what was predicted based on the modeled pilot data in Phase 1. This may be the result of the shorter response format and item numbers creating less variance in responses. In both the validation survey (Phase 2) and beta testing (Phase 3), Social, Foodie, and Overthinking styles were the dominant styles, and a dominant Impulsive style was much less common. Furthermore, two items from this construct fell below recommended values for strong construct validity. The decision was made to retain these items to maintain balance in the number of items across the survey. Most of the users of the live website (Phase 3) agreed with the dominant style they were presented with. Thus, based on the above metrics, it appeared that the development of a short-form Diet Styles Survey was scientifically solid and acceptable to consumers.

## 3 Real-world launch and uptake (Study 2)

The second aim of this research was to develop, launch, and evaluate a real-world, consumer-facing tool to understand its possible engagement and interest and to test the different segmentations.

To ensure its relevance and appropriateness for the public, the final shortened items and presentation of results were co-designed between the commercial partner and research team. The final consumer-facing tool included the short form Diet Styles Survey and the presentation of individuals’ dominant diet style with easy-to-read information about each diet style. The development of the items was finalized by the research team prior to this process, and the commercial partner had no input into the selection of the survey items or composition of the Styles. The partner contributed their market expertise and knowledge gained from in-house AB testing of existing offerings to help with the presentation of information in an accessible and appealing way. To ensure scientific integrity, the research team gave final approval of any proposed changes.

As a result of this codesign process, the tool was renamed “Diet Types,” and the original names of the diet styles were changed from Overthinking to Thinker; Temptation to Craver; Social to Socializer; Impulsive to Freewheeler; and Foodie remained the same (described in Table 1). Most of the name changes reflected an attempt to reduce any negative connotation or perceived judgment. For example, ‘overthinking’ implies an imbalance, and therefore Thinker was chosen as it was similar to the original name without the possible judgment. The Temptation Style was constructed by items from a Craving inventory, and the shift in name in this instance was scientifically appropriate, as well as being viewed as more appealing to consumers. The change of Impulsive to Freewheeler took the most iterations and again focused on minimizing any negative connotation. There was discussion about reframing the term to capture control as the flipside to impulsivity, but given active debate about the singularity versus duality of control and impulse, it was essential that the label reflected this trait more closely. All changes were piloted by broader members of the partner and research teams to ensure fit scientifically as well as comprehensibility.

**Figure 1:**
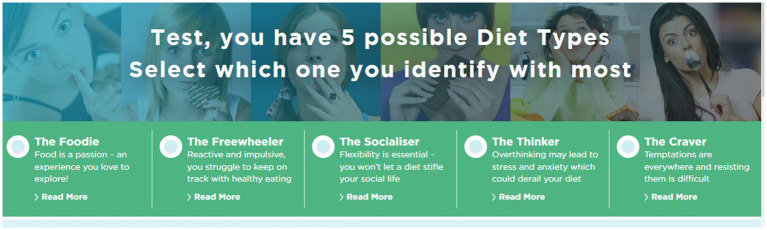
Example screenshot of feedback for a user with equal scores across all 5 possible Types. Reprinted with permission from Digital Wellness.

Based on the development and validation study observations, it was predicted that less than half of the sample would have a single dominant diet type. Therefore, a feature was included as part of the consumer tool where respondents without a single dominant type were presented with their top types and prompted to self-select a preferred diet type (Figure 1 shows a person with equal scores across all 5 domains).

### 3.1 Method

The Diet Types tool was uploaded to the homepage of a commercial weight loss program (the CSIRO Total Wellbeing Diet online; https://www.totalwellbeingdiet.com/ should work totalwellbeingdiet.com.au). The launch of Diet Types occurred in January 2017, and the tool was promoted through television, print, and social media stories. Interested participants were directed to the website where they could find and complete the tool of their own volition. The Diet Types Survey took less than 10 min to complete. Data were collected and stored in Survey Gizmo and analyzed descriptively to understand uptake and the type of people whom the tool appealed to relative to the general Australian population, the most common dominant diet types, and any differences by demographics between the different diet types.

### 3.2 Results

**Figure 2:**
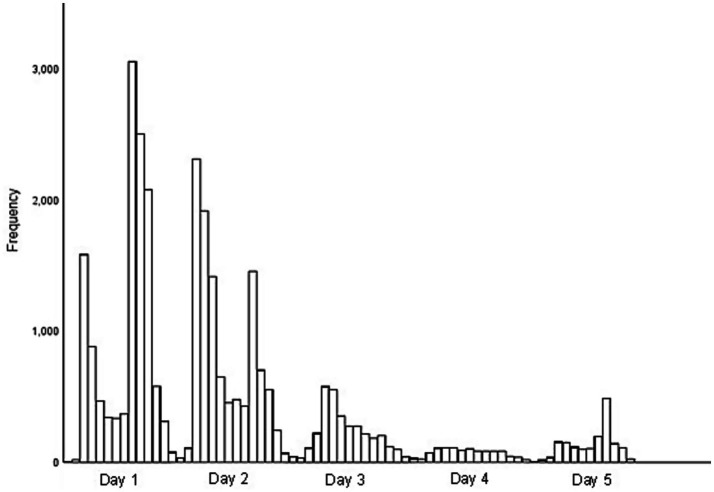
Complete responses to the Diet Types Survey in the first 5 days following media launch, total *n* = 29,394.

Within 5 days of the launch of Diet Types, the survey had received 29,394 valid responses (Figure 2).

Those completing the survey were mostly women (81.3%), aged 30–49 years (42.9%) or 50–69 years (36.0%). Three-quarters of the sample resided in the eastern states of Australia (New South Wales, Victoria, and Queensland) and were categorized as overweight or obese (41.9% obese) according to BMI (see Table 4).

**Table 4:** Participant demographic characteristics for those completing Diet Types within the first 5 days of its launch in 2017, compared to Australian population data.

Variable	*N*	% (valid)	% (Aust pop)*
Sex (female)	23,902	81.3	50.7
Age group (years), 1 missing			
19–29	5,388	18.3	13.8
30–49	12,621	42.9	27.5
50–69	10,571	36.0	23.4
70+	812	2.8	10.6
State of residence			
Victoria	8,297	28.2	25.3
New South Wales	7,433	25.3	32.0
Queensland	5,890	20.0	20.1
Western Australia	3,495	11.9	10.6
South Australia	2,308	7.9	7.2
Australian Capital Territory	940	3.2	1.7
Tasmania	617	2.1	2.2
Northern Territory	413	1.4	1.0
Weight status (BMI), 489 missing			
Underweight	318	1.1	1.3
Normal	7,258	25.1	31.7
Overweight	9,223	31.9	35.6
Obese	12,106	41.9	31.1

*Australian population (Aust pop) data based on 2017.0 census.... and weight status from 4364055001DO008_.

2071.0 Census of population and housing: reflecting Australia—stories from the census, 2016—snapshot of Australia.

Relative to the Australian population data, this sample had disproportionate numbers of females and those between 35 and 50 years old. Based on National Health Survey results for 2017/18, the sample also had an overrepresentation of those classified as obese according to BMI (41.9% vs. 31.1%). Approximately 45.7% Australians recorded an occupation in 2016 based on employment data from the Australian Census. In comparison, 78.6% of participants reported an occupation in the responses received.

One third of the sample (33.8%, *n* = 9,935) were classified as having a single dominant type, with an additional 38.5% (*n* = 11,310) having two equally dominant options and 21.0% (*n* = 6,179) having three equally dominant types. Approximately one-quarter of the sample (*n* = 8,137; 27.7%) were presented with multiple types but failed to select one when prompted.

Of the sample with a single dominant type, most (*n* = 7,123; 72%) provided feedback on whether they thought that the type presented suited them, and of these, 97.0% (*n* = 6,909) agreed with the description provided. Acceptance was above 90% for all Types. Of those who disagreed with their types (*n* = 214), there were no clear themes in comments received, although there was a suggestion made by more than one about the inaccuracies in specific statements used to describe the Type.

The selected or agreed Diet Type was stored in the system. Among the stored data (*n* = 21,257), Thinker was the most common dominant type (29.7%), followed by Craver (18.1%) (Table 5).

**Table 5:** Number of participants in each Diet Type category based on final user selections.

Diet Type	Full sample			Single Type only	
	*n*	%	Adjusted %	*n*	%
Socializer	3,100	10.5	14.6	1,284	12.9
Craver	5,311	18.1	25.0	1966	19.8
Freewheeler	912	3.1	4.3	115	1.2
Foodie	3,212	10.9	15.1	1,649	16.6
Thinker	8,722	29.7	41.0	4,921	49.5
Unknown	8,137	27.7	–	–	–

Unknown indicates participants with multiple high scores who failed to select a Type.

Differences between diet types based on demographic characteristics were like those seen in the previous development studies. There were more women classified in the Thinker and Craver types (Table 6). Over half of the Craver and Freewheeler Types were classified as living with obesity according to BMI. The Foodie Type had the highest proportion of normal weight people as well as those who had never dieted (Table 6).

**Table 6:** Diet Type by participant characteristics, including sex, age, weight status based on Body Mass Index (kg/m^2^), and self-reported lifetime diet attempts (*n* = 29,934).

Variable	Diet Type (%)					Total
	Thinker	Craver	Foodie	Socializer	Freewheeler	
Sex *n* = 21,257						
Female	85.2	82.6	75.8	78.3	69.7	81.5
Age group *n* = 21,256						
19–29	16.6	17.2	14.3	17.0	14.4	16.4
30–49	43.7	44.7	41.7	37.3	45.4	42.8
50–69	37.1	35.6	40.7	41.0	37.2	37.9
70+	2.5	2.4	3.3	4.6	3.0	2.9
Weight status (BMI) *n* = 20,924						
Underweight	1.1	<1	<1	<1	<1	<1
Normal	26.9	14.2	28.5	26.2	14.3	23.4
Overweight	32.3	28.7	35.1	35.4	31.0	32.2
Obese	39.7	56.6	35.5	37.5	54.1	43.5
Lifetime diet attempts *n* = 21,216						
Never	7.2	3.3	10.2	9.4	8.9	7.1
1–5 times	42.9	36.5	48.6	46.1	42.9	42.9
6–10 times	21.6	23.8	19.8	20.8	20.4	21.6
11–15 times	9.0	10.8	7.6	7.9	9.0	9.0
16–25 times	5.5	6.7	4.2	4.9	5.5	5.5
More than 25 times	13.9	18.9	9.5	10.9	13.2	13.9

### 3.3 Conclusion from real world launch

There was high interest and participation with the Diet Types tool, with almost 30,000 people completing the survey within 5 days of its launch. Those interested were not representative of the wider Australian population, with the tool attracting a sample with a greater proportion of females, those with larger body sizes, and those in employment than the Australian public generally.

The structure of the tool and the association between Diet Types and participant characteristics replicated those seen in the beta phase. There were more Freewheelers and Cravers classified as living with obesity, and more Cravers with high numbers of dieting attempts. Refinements to the user experience will be needed to reduce the number of participants failing to select a Diet Type, and to better understand whether this is a system design challenge or a failing of the five Diet Types to capture these respondents.

## 4 General discussion

This study has reported on the development and testing of a consumer-facing tool designed to segment individuals and provide information about their diet style. Reducing the number of items to a length more acceptable for a consumer-facing segmentation tool through beta testing and a stepwise approach facilitated the development of a final product with high uptake by the Australian public. This approach was pragmatic and designed to be applied. Consequently, there is no way of knowing whether the item reduction, co-design, or general appeal of the final tool was a key pillar of this engagement.

The Diet Types tool characterized people into one of five segments, with the two most common diet types being Thinker and Craver. Following the launch of the tool, Thinker was the most common diet type, which was different from the most frequent Type observed in development studies. Thinker originally included neuroticism and behavioral inhibition. Neuroticism is thought to be on the increase in the population (Twenge, 2000) and is higher in females relative to males (Jorm, 1987). The samples in this study included higher numbers of females, which could explain the dominance of this type. Overthinking was also associated with lower ratings of perceived health in our initial study (Brindal and Golley, 2021). Dwelling on health could be one of the traits associated with this diet type, and this may extend to weight management and eating. However, it is unclear whether greater concern translates into poorer outcomes or whether this disposition leads people to be more focused on their general health. Interestingly, people who disagreed with the diet type they were presented with were also happy to self-select Thinker (data not presented).

Across all studies, Cravers stood out as a distinct segment with higher BMI, more dieting attempts, higher concern over their current weight, and lower perceived success at maintaining weight. Cravings have been linked to disinhibition, overeating, and loss of control (Whatnall et al., 2022) and emotional eating (Verzijl et al., 2018). There are many programs that can target cravings with varying degrees of success, and directing people to these kinds of interventions might be helpful for weight management (Alberts et al., 2010; Hamilton et al., 2013; Jansen et al., 2016). Many of these focus on the mental imagery and visualization processes associated with food cravings. Being able to identify segments that experience high levels of cravings may assist in targeted assistance in weight management in an area where practical interventions exist.

Throughout each stage of this process, the Impulsive/Freewheeler Style represented a small subgroup, but the type was retained, given its validity as a separate construct which remained independent and discrete as a type. If a few people are dominant in this as a type, it is possible that this adds minimal value to the overall segmentation. One of the reasons that this diet type fails to be identified as dominant relative to others is because of lower levels of agreement with the items capturing this type. Characteristics underpinning impulsivity may be seen as deficits, particularly when these traits are commonly included in disorders labelled attention-deficit and researched in relation to psychopathy (e.g., Eisenbarth et al., 2008). There is good evidence that non-neutral traits can distort self-perception (McCrae and John, 1992). Craving, while also a possible deficit, may be more socially palatable and understood; therefore, items associated with this type may score more highly in terms of agreement. The Impulsive/Freewheeler diet type was initially retained for its potential importance in eating behaviors and the possibility of tailoring intervention and advice based on this characteristic (Brindal and Golley, 2021). In future, the Freewheeler type could be removed or used to classify people into subtypes, for example, Cravers with high or low impulsivity.

A long history of personality assessments has opted for approaches that either classify people into profiles (Briggs, 1987) or define traits (Eysenck and Eysenck, 1976). Academically, there appears to be stronger support for defining traits because it reduces limitations created by, for example, arbitrary cut-offs for particular profiles. Creating profiles can also lead to controversial dichotomizing in terms of construct validity. For example, despite acceptance of extraversion and introversion as core dispositional traits, there has been debate about whether these are types and whether they belong on a single axis (Guilford and Braly, 1930; McCrae and Costa Jr, 1989). High use of tools assessing the Big 5 (McCrae and John, 1992) suggests that researchers and academics prefer to use trait tools.

In practice, consumer-facing tools tend to favor typing tools because they can place people into types and segments that offer simpler tailoring of feedback. For example, there are many commercial or consumer-facing tools that are iterations of Jung’s personality typing tool with surveys based on the Myers-Briggs Type Indicator, enjoying wide engagement (e.g., 16 Personalities has millions of users) or managerial application (Schweiger, 1985; Pittenger, 2005) and commercial success in organizational assessments of management profiles.^1^

There is little doubt that scales provide richer potential for the exploration of data. We opted to present a dominant type that is neither a single trait nor a profile. This typing approach worked for classifying roughly a third of people across our samples, which suggests that having a single dominant type may not be the optimal approach. Adding more traits to each type could assist in the development of something resembling more of a profile. Participant feedback from this study largely indicated that people were happy with the approach used. However, no empirical data were collected that explicitly tested different presentation methods. Despite scientific and statistical limitations of type-based approaches, the perceived value of simplification for the broader community cannot be discounted. The ability of the tool to ultimately segment people to allow tailored materials also requires a similar classification method to determine who will see what. The full quantitative data are stored and available for analysis and scientific exploration. It is therefore possible to use the final responses in both ways simultaneously. Given the apparent interest in the tool, it could also be useful in future to explore offering a more nuanced profile that shows levels as well as a dominant type.

Co-designed research is experiencing a resurgence, particularly in health, to largely describe higher levels of patient engagement and consultation (Slattery et al., 2020). Our research design was one with multiple layers of engagement from the public as well as a commercial partner. The development of this tool was led mostly by science, and public feedback was sought regarding its face validity. More in-depth co-design occurred with our commercial partner, which at times was challenging but allowed us to push our science into a highly engaged consumer-facing tool and achieve reach far beyond that of other tools. It was important that during this process, the science team was given time to reflect on changes and their implications based on existing scientific knowledge, which at times meant re-reviewing literature and theories. Similarly, the research team was receptive to suggestions.

People have implicit theories about personality types and prototypes about other people, which can be insightful (Schneider, 1973). Some people shared suggestions for prototypes beyond those captured. A small number of responses indicated further interest from the public in healthy and emotional eating types. Eating for emotional reasons initially loaded onto the Craver diet type, and the description of this could include greater emphasis on this component in future. In terms of a type capturing healthier eaters, there is good evidence that clusters of healthier eaters can be identified (Newby and Tucker, 2004). However, shifting toward a type based on meeting dietary recommendations could incidentally capture orthorexia (Yakin et al., 2021). This would require more detailed analysis of people’s dietary intake, removing focus away from disposition and behavior, and lengthening the survey. Finally, results indicate that there are behavioral differences between types, and therefore, dietary and activity behaviors have been classified as outcomes rather than components of these types. Future studies could look to more comprehensively evaluate the types against lifestyle behaviors.

### 4.1 Strengths and Limitations

Despite high participation numbers, the tool, as well as all the development surveys, attracted a disproportionality higher number of female participants. In paid public health campaigns relying on media in Australia, women are slightly overrepresented but not to the extent seen here (O'Hara et al., 2012; Smith et al., 2006). Women are reported to have stronger interest and engagement in healthy eating and weight management (Wardle et al., 2004). In some ways, this means that this tool has reached the primary target of those engaged with weight management, which is a strength. However, it also means that it may not be successful as a tool for reaching audiences with lower existing interest or engagement. In terms of public health, engaging males in diet and weight management research presents an ongoing challenge, but an important challenge given the higher prevalence of obesity. It is possible that other tools will be needed to reach these groups, or a modified version of this tool. The limited representation also means that the Diet Types may not be particualrly male-orientated or reflective of younger audiences which are likely to be absent from the final tool. For example, it remains unclear if the Freewheeler type is more dominant in males or whether other types are not capturing behaviors relevant for men.

In the interest of shortening the tool, only a single trait for each Type was captured in the final version of the survey. This was the trait that most strongly represented the underlying construct and has a method used previously (Maloney et al., 2011). However, during the refinement and development of this tool, we failed to evaluate how well it related back to the larger, multifaceted styles originally reported. Future work will need to address this gap.

All surveys were self-report, and while this appears appropriate for personality assessment, there are many studies that seek to assess the accuracy of measures relative to the feedback of others (e.g., Allik et al., 2015; McCrae, 2008). Further understanding the validity of these types may require assessment of profile agreement between different observers.

Finally, despite significant progress towards developing a simple tool facilitating tailored messages that could be useful for segmentation and implemented into an existing program, the current research did not assess the efficacy of tailored messages for engagement or weight loss. Other factors beyond segmentation, such as personal relevance and actionable advice, also need to be considered in effective tailoring (Lustria et al., 2013).

## 5 Conclusion

There is often perceived tension between the rigorous scientific process and public engagement. The Diet Types Survey was developed through a systematic process that involved direct feedback from participants and workshopping with the commercial partner to optimize its appeal. Balancing stakeholder feedback can be challenging, and at times, scientific rigor was compromised for consumer appeal. For example, the dramatic reduction in questions meant losing assessment of multiple facets of each Type. Despite these limitations, the final diet type tool was developed incrementally in a replicable process that involved large samples of data. Furthermore, the uptake of the tool in its first few days provides an indication of interest in this tool from the Australian public, particularly older women living with obesity. In future, we hope to make further refinements to weight management programs to provide tailored advice around these diet types and evaluate if this approach to segmentation can be used to influence real-world data.

## Data Availability

The raw data supporting the conclusions of this article are not readily available and are subject to ethical approval. Requests to access the datasets should be directed to the corresponding author.

## Footnotes

1
https://www.teammanagementsystems.com/our-products/
